# Characterizing nucleosome dynamics from genomic and epigenetic information using rule induction learning

**DOI:** 10.1186/1471-2164-10-S3-S27

**Published:** 2009-12-03

**Authors:** Ngoc Tu Le, Tu Bao Ho, Dang Hung Tran

**Affiliations:** 1 School of Knowledge Science, Japan Advanced Institute of Science and Technology, 1-1 Asahidai, Nomi, Ishikawa 923-1292, Japan; 2 Hanoi National University of Education, 136 Xuan Thuy, Cau Giay, Hanoi, Vietnam; 3 Vietnamese Academy of Science and Technology, 18 Hoang Quoc Viet, Cau Giay, Hanoi, Vietnam

## Abstract

**Background:**

Eukaryotic genomes are packaged into chromatin, a compact structure containing fundamental repeating units, the nucleosomes. The mobility of nucleosomes plays important roles in many DNA-related processes by regulating the accessibility of regulatory elements to biological machineries. Although it has been known that various factors, such as DNA sequences, histone modifications, and chromatin remodelling complexes, could affect nucleosome stability, the mechanisms of how they regulate this stability are still unclear.

**Results:**

In this paper, we propose a novel computational method based on rule induction learning to characterize nucleosome dynamics using both genomic and histone modification information. When applied on *S. cerevisiae *data, our method produced totally 98 rules characterizing nucleosome dynamics on chromosome III and promoter regions. Analyzing these rules we discovered that, some DNA motifs and post-translational modifications of histone proteins play significant roles in regulating nucleosome stability. Notably, these DNA motifs are strong determinants for nucleosome forming and inhibiting potential; and these histone modifications have strong relation with transcriptional activities, i.e. activation and repression. We also found some new patterns which may reflect the cooperation between these two factors in regulating the stability of nucleosomes.

**Conclusion:**

DNA motifs and histone modifications can individually and, in some cases, cooperatively regulate nucleosome stability. This suggests additional insights into mechanisms by which cells control important biological processes, such as transcription, replication, and DNA repair.

## Background

Genetic materials of eukaryotic organisms are packaged into chromatin inside cell nucleus. This compact structure has the form like a bead-on-string fiber containing fundamental repeating units, the nucleosomes. Each nucleosome is composed of 147 bp of DNA wrapped 1.65 turns around an octamer of histone proteins consisting of a central (*H*3 - *H*4)_2 _tetramer flanked on both side by two *H*2*A *- *H*2*B *dimers [1]. Since it was first recognized [2], there have been increasing evidences showing that chromatin plays a much more important role far beyond DNA compaction. By burying *cis*-regulatory elements under histone proteins and/or modifying related epigenetic information, chromatin imposes ubiquitous and profound effects on many DNA-based processes, including transcription, DNA repair and replication. To ensure faithfully copy both genetic and epigenetic information during replication or to facilitate the binding of Transcription Factors (TFs) to regulatory elements during transcription in the context of chromatin, cells have developed complicated biological pathways [3]. In these pathways, by regulating nucleosome stability cells can control the accessibility of underlying DNA sequences to biological machineries. For example, in replication, during the process known as parental histone segregation, pre-existing nucleosomes located ahead of replication forks are transiently disrupted from parental DNA strands and later transferred onto nascent DNA [3,5]. In transcription, moving nucleosomes to different translational positions is known as one way to change the accessibility of nucleosomal DNA to TFs [4]. Also, promoter regions of actively transcribed genes are usually free of nucleosomes [7,8]. So, understanding how cells regulate nucleosome stability will bring us additional insights into mechanisms of many important biological processes.

Nucleosome stability can be regulated by many factors, such as DNA sequences, histone modifications and histone variants, and chromatin remodelling complexes [9]. For example, DNA sequence is known as a reliable determinant for nucleosome preference, which can be used to predict nearly 50% of nucleosome positions [10], so it is likely to be an important factor in favouring or disfavouring nucleosome eviction. Histone variant H2A.Z (Htz1) is found to be preferentially enriched at promoters where some nucleosomes have to be quickly removed upon transcriptional activation [4]. Also, acetylated histones are shown to be easily dissociated from DNA [11,12]. Chromatin remodelling complexes, such as Swi/Snf, act in concert with histone chaperones (e.g Asf1, Nap1) to displace histones from their original positions [4]. Although the complete list of factors has been fairly known, the mechanisms of how they act to mobilize nucleosome are still unclear.

Owing to recent advanced profiling techniques, such as ChIP-on-Chip and ChIP-Seq, we now have increasing amount of information about how nucleosomes and various kinds of histone modifications are distributed over the genomes of many organisms, including yeast, drosophila, and human [13,8]. This opens up a chance for thorough investigation of nucleosome organization, its regulatory mechanisms and functions. Until now, there have been many works, both experimental and computational, concentrating on revealing the effects of factors stated above on nuclesome distribution [10,13,19,20] but most of them have some common drawbacks. First, they mainly considered the effect of each factor separately while bypassing their combinatorial effects on nucleosome distribution. Second, although the distribution of destabilized nucleosomes is usually inhomogeneous throughout the genome and is known to have strong relation with transcriptional activities [13], it is still not well-characterized compared with that of stable nucleosomes.

There are several efforts trying to overcome these limitations. For example, Rippe et al. [21] and Schnitzler [22] investigated co-effects of DNA sequences and chromatin remodelling complexes; Widlund et al. [23] and Yang et al. [24] investigated co-effects of histone tails and DNA sequences on nucleosome distribution. Most of them, however, were based on experimental methods. More recently, Dai et al. [25] used both transcriptional interaction and genomic sequence information to computationally identify dynamic nucleosome distribution, but the number of works like this is still limit.

Enthused by these facts, in this paper, we propose a novel method for computationally characterizing nucleosome dynamics from both genomic sequences and histone modification profiles. Our method is based on induction rule learning adapted for subgroup discovery, which can discover sufficiently large and statistically meaningful subsets of population as shown in [26], so it is well suited for characterizing inhomogeneous distribution of destabilized nucleosomes. Moreover, by combining both genetic sequence and histone modification information, our method can discover the combinatorial nature of these two factors in regulating nucleosome stability. Our results on *S. cerevisiae *show that, some DNA motifs, which are reliable determinants for nucleosome forming/inhibiting potential, and post-translational modifications of histone proteins, which have strong relation with transcriptional activities, are likely to be more significant to nucleosome dynamics. We also found some patterns of cooperation between these DNA motifs and histone modifications in regulating nucleosome stability. Our results give additional insights into mechanisms of how cells regulate important biological processes, such as transcription, DNA repair and replication.

## Results and discussion

### Potentially significant motifs to nucleosome dynamics

DNA sequence has long been known to be a strong determinant for nucleosome formation potential, which can be used to identify nearly 50% of positioned nucleosomes *in vivo*, so it is likey to be an important factor affecting nuclesome stability. To determine DNA motifs which may be importantly related to nucleosome stability, two different approaches were applied (Section *Method overview*). In the first one, we used WordSpy [27] with the word length set to 6 to identify statistically significant motifs related to nucleosome states. The length of 6 was chosen because, as shown in some previous research [10,19], nucleosome forming ability of DNA sequences may be decided mostly by short motifs, with length from 2 to 6. WordSpy uses dictionary-based approach so it is suitable to find short motifs among a group of DNA sequences [28]. Tables 1 and 2 show the 15 most significant motifs related to 2 states of nucleosomes found by WordSpy when run on chromosome III and promoter region data, respectively (complete lists are given in Additional File 1 and 2). The results show no big difference between important motifs of genetic regions and those of promoter regions. For example, both of them are enriched of dinucleotides TG/CA and this coincides with previous research [19], showing that TG/CA are highly flexible dinucleotides so they have large impact in imparting nucleosome forming ability. From the results given by WordSpy, it is difficult to identify motifs that may be important in discriminating nucleosome states. So, we used the second approach based on feature selection with Fisher criterion (Section *Feature selection with Fisher criterion*) to overcome this limitation. Table 3 shows 20 strongest discriminative motifs corresponding to chromosome III and promoter regions ranked by their F-score values (complete list is given in Additional File 3). Among them, dinucleotides are likely the most important motifs compared with the others in deciding nucleosome stability: 14 and 15 over 20 in chromosome III and promoter sequences, respectively. Moreover, among 10 strongest discriminative signals are AA/TT/AT/TA/CA/TG (for chromosome III) and AT/TT/CA/TG (for promoter regions), which are related with nucleosome forming (e.g. CA/TG) and inhibiting (e.g. AA/TT/AT/TA) potential of DNA sequences.

**Table 1:** Significant DNA motifs on chromosome III given by WordSpy

	*Well-positioned*				*Delocalized*			
Order	Motifs	ZScore	Occur#	Seq#	Motifs	ZScore	Occur#	Seq#
1	TG	12.9	10778	997	TG	6.6	2690	154
2	CA	12.6	11461	997	CAA	10.3	1128	153
3	TTC	17.3	3917	963	TTG	9.8	953	153
4	TGG	15.7	2485	878	GAA	9.4	1064	154
5	GAA	15.5	3822	956	CCA	9.2	735	151
6	CCA	15.4	2728	902	TTC	9.0	967	151
7	CTTC	17.2	1023	581	TGG	7.7	615	148
8	TTTC	15.3	1349	699	TTTG	10.1	350	139
9	TTCT	13.7	1311	675	CTTC	9.4	259	105
10	TTTG	13.4	1247	696	GAAA	8.6	390	135
11	GAAG	13.3	893	554	GAAG	8.0	253	114
12	CCAA	12.5	937	581	TTCT	7.2	321	132
13	AAGA	12.2	1290	645	AAAG	6.7	360	135
14	TGGA	11.5	830	534	AGAA	6.7	362	136
15	AGAA	11.5	1268	665	TCTTC	10.3	111	64

ZScore is computed by using WordSpy.

Occur# is the number of occurrences of the DNA motif in DNA sequences.

Seq# is the number of sequences containing the DNA motif.

**Table 2:** Significant DNA motifs on promoter regions given by WordSpy

	*Well-positioned*				*Delocalized*			
Order	Motifs	ZScore	Occur#	Seq#	Motifs	ZScore	Occur#	Seq#
1	TG	11.4	10865	995	TG	3.7	1164	69
2	CA	10.4	10913	995	TTG	5.7	406	66
3	GC	4.7	7254	992	TTC	5.3	400	67
4	GA	4.6	10360	993	TGG	4.7	286	61
5	CAA	14.9	3707	949	AGA	4.6	377	67
6	GAA	14.8	3696	948	CAA	4.5	371	69
7	TTC	13.6	3576	954	TTTC	5.3	141	52
8	TGG	12.6	2552	897	GGAA	5.1	101	48
9	CCA	10.5	2493	909	TTCTT	9.9	79	38
10	CTG	8.6	2384	897	TCTTC	7.5	52	34
11	TCT	8.2	3323	926	TTTCT	7.4	65	36
12	TTTG	14.1	1239	720	CTTCT	7.1	50	35
13	TTTC	14	1237	692	TCTTT	6.1	58	35
14	CTTC	13.2	910	553	AGGAA	5.8	42	31
15	CTTT	13.2	1216	668	AAGAA	5.6	53	39

ZScore is computed by using WordSpy.

Occur# is the number of occurrences of the DNA motif in DNA sequences.

Seq# is the number of sequences containing the DNA motif.

**Table 3:** Discriminative motifs ranked by F-scores

	*Chromosome III*		*Promoter Regions*	
Order	Motifs	F-score	Motifs	F-score
1	AT	1.37683	AG	0.69706
2	CA	1.12833	CT	0.623328
3	GA	0.913882	TG	0.577693
4	TG	0.894409	GA	0.575111
5	AA	0.882082	AT	0.572648
6	TA	0.813029	GC	0.537435
7	AG	0.811749	TC	0.517756
8	AC	0.803107	CA	0.507869
9	AAT	0.741735	GT	0.483424
10	TT	0.736747	TT	0.455674
11	CT	0.68323	CTT	0.452965
12	TC	0.64163	TA	0.446487
13	GT	0.615279	AA	0.41366
14	CAA	0.574223	AC	0.381596
15	GAA	0.523384	GAG	0.367994
16	GC	0.501134	GG	0.363897
17	ATT	0.499311	CC	0.362195
18	TAA	0.477322	TTC	0.330391
19	CC	0.455241	TAG	0.329403
20	TGA	0.453114	ATT	0.32476

### Significant histone modifications to nucleosome dynamics

Histone modification is one of the most important non-sequence regulatory factors of many chromatin-based processes and has also been known to affect nucleosome stability. To identify histone modifications potentially significant to nucleosome stability, we applied feature selection procedure, the same as what was done with DNA sequences, on the data of 12 different histone modifications corresponding to chromosome III and promoter regions (Section *Data preparation*). The result was ranked by F-score and given in Table 4. This result shows that, the first 9 modifications of chromosome III, including H3K14Ac/H4K5Ac/H3K4Me3/H4K12Ac/H3K4Me1/H3K9Ac/H2AK7Ac/H4K16Ac/H2BK16Ac, and the first 6 ones of promoter regions, including H3K4Me3/H3K9Ac/H3K18Ac/H4K16Ac/H4K12Ac/H4K8Ac, seem to be more important to nucleosome stability. Notably, all significant modifications in promoter regions are strongly related to transcriptional activation (e.g. H3K4Me3/H3K9Ac/H3K18Ac) and repression (e.g. H4K16Ac/H4K12Ac/H4K8Ac) [17,18,29]. That is also true with some significant modifications in chromosome III, where H3K4Me3/H3K9Ac and H4K12Ac/H4K16Ac/H2BK16Ac are known to have strong relation with transcriptional activation and repression, correspondingly.

**Table 4:** Histone modifications ranked by F-scores

	*Chromosome III*		*Promoter Regions*	
Order	Modifications	F-score	Modifications	F-score
1	H3K14Ac	0.102054	H3K4Me3	0.0328115
2	H4K5Ac	0.0863558	H3K9Ac	0.0322587
3	H3K4Me3	0.0754543	H3K18Ac	0.0315715
4	H4K12Ac	0.0660357	H4K16Ac	0.0253305
5	H3K4Me1	0.0586061	H4K12Ac	0.0230635
6	H3K9Ac	0.0398707	H4K8Ac	0.0229266
7	H2AK7Ac	0.0309521	H3K4Me1	0.00913233
8	H4K16Ac	0.0219245	H2AK7Ac	0.00767291
9	H2BK16Ac	0.019511	H4K5Ac	0.00318472
10	H3K18Ac	0.00603551	H3K4Me2	0.00283706
11	H3K4Me2	0.004844	H2BK16Ac	0.00022866
12	H4K8Ac	9.68E-06	H3K14Ac	9.89E-06

### Effects of DNA sequences and histone modifications on nucleosome dynamics

In order to see how DNA sequences and histone modifications affect nucleosome stability, we applied our method to the data containing significant DNA motifs and histone modifications identified above (Section *Method overview*). After filtering out uninteresting rules (Section *Rule filtering*), we received two sets of 60 rules (given in Additional File 4) and 38 rules (given in Additional File 5) characterizing nucleosome dynamics on chromosome III and promoter regions, correspondingly. Table 5 shows some selected rules from these rule sets. Analyzing these rules, we discovered that the enrichness of some specific DNA motifs has special impact on nucleosome stability. For example, nucleosomes bound by sequences enriched with AT/ATT/CTT are more stable (rules 1, 2, 6, 9, 10). This agrees with the result from [19], which said that sequences enriched with dinucleotides AT/TT have potential to inhibit nucleosome forming and deforming them on nucleosomes is more costly, so nucleosomes bound by these sequences may be more stable. Also, H3K9Ac/H3K18Ac/H3K4Me3 are known to have positive relation with transcriptional activation [17,18,29], so nucleosomes which are hyper-acetylated at H3K9/H3K18 and hyper-trimethylated at H3K4 seem to be more dynamic (rules 7, 8). In contrast, H4K12Ac is known to have positive relation with transcriptional repression [29], so H4K12 hyper-acetylated nucleosomes are more stable (rule 5) while H4K12 hypo-acetylated nucleosomes are more dynamic (rules 11, 12). However, there is no DNA pattern or post-translational modification showing dominant effect on nucleosome stability. Instead, there exist combinatorial effects, by DNA motifs themselves (rules 3, 4, 9) or by both DNA motifs and histone modifications (rules 2, 5, 7, 8, 10, 11, 12), on nucleosome stability. For example, if H3K4Me3 or H3K9Ac nucleosomes are located in regions enriched with ATT tri-nucleotide, they will become more stable (rules 2, 10); and even being located in regions enriched with AT dinucleotide, H4K12 hypo-acetylated nucleosomes still have potential of becoming unstable (rule 12). This agrees with the results from previous and recent works showing that the effects of histone acetylations depend on which lysines are acetylated and the locations of modified nuclesomes [30,2]; and nucleosome positioning effect of DNA sequences is decided by the combination of nucleosome favouring and disfavouring motifs [19,33].

**Table 5:** Selected rules characterizing nucleosome dynamics

**No**.	Rules	**Class dist**.
1	*AA*, *ATT *= *enr *∧ *H*3*K*9*Ac *= *neutral *→ *State *= *Well*	[300 0]
2	*ATT *= *enr *∧ *H*3*K*4*Me*3 = *hyper *→ *State *= *Well*	[156 0]
3	*AT*, *GC *= *enr *∧ *CC *= *low *→ *State *= *Well*	[159 0]
4	*AT, CC *= *enr *∧ *GC *= *low *→ *State *= *Well*	[56 0]
5	*AT *= *low *∧ *H*3*K*9*Ac *= *neutral *∧ *H*4*K*12*Ac *= *hyper *→ *State *= *Well*	[10 0]
6	*AT*, *TC *= *low *∧ *ATT *= *enr *→ *State *= *Well*	[13 0]
7	*CT*, *TG*, *GA*, *AT*, *CTT*, *GAG*, *ATT *= *low *∧ *H*3*K*18*Ac*, *H*3*K*4*Me3 *= *hyper *→ *State *= *Del*	[0 6]
8	*GA*, *TT*, *GG *= *low *∧ *H*3*K*9*Ac *= *hyper *∧ *H*3*K*4*Me3 *= *hypo *→ *State *= *Del*	[0 3]
9	*AA *= *low *∧ *GT*, *ATT *= *enr *→ *State *= *Well*	[77 0]
10	*ATT *= *enr *∧ *H*3*K*9*Ac *= *hyper *→ *State *= *Well*	[66 0]
11	*GA*, *AG*, *ATT *= *low *∧ *H*2*BK*16*Ac *= *neutral *∧ *H*4*K*12*Ac *= *hypo *→ *State *= *Del*	[0 15]
12	*AT *= *enr *∧ *TA*, *TAA *= *low *∧ *H*3*K *9*Ac *= *neutral *∧ *H*4*K*12*Ac *= *hypo *→ *State *= *Del*	[0 4]

*enr*, *Well *and *Del *are the abbreviations for *enriched*, *Well-positioned *and *Delocalized*, correspondingly.

## Conclusion

Nucleosome dynamics plays important roles in many DNA-based processes and is regulated by many factors, such as DNA sequences, post-translational modifications of histone proteins, and chromatin remodelling complexes. However, most of the previous works only investigated the effect of individual factor while bypassing their combinatorial effects on the distribution of stable nucleosomes. In this paper, we proposed a novel method based on induction rule learning to computationally characterize nucleosome dynamics from both genomic and histone modification information. Our method is shown to be suitable for characterizing inhomogeneous distributions like that of destabilized nucleosomes; and by combining both genomic and histone modification information, it can discover potential co-effects of these two factors on nucleosome dynamics.

Our results on *S. cerevisiae *show that, some DNA motifs and histone modifications are more important in stabilizing and destabilizing nucleosomes. These DNA motifs and histone modifications are known to have strong relations with nucleosome forming/inhibiting potential and transcriptional activities, correspondingly. They not only act individually but also cooperate with each other by some specific patterns to combinatorially affect nucleosome stability.

Although our method is efficient in characterizing nucleosome dynamics, it produces a larger number of rules, of which many may be irrelevant. In the future, we need to develop a better method for filtering these uninteresting rules.

## Methods

### Data preparation

We used experimental data from Yuan et al. [13] and Liu et al. [17], which covered nearly 4% of yeast genome including chromosome III and 223 additional promoter regions, for our experiments. Data from Yuan contained 50-base DNA fragments tiled every 20 base pairs, and for each fragment we extracted its genomic sequence and HMM inferred state showing that it is nucleosomal sequence or not. Data extracted from Liu contained 12 different histone modification levels corresponding to DNA fragments above, including acetylations of H3K9, H3K14, H3K18, H4K5, H4K8, H4K12, H4K16, H2AK7, H2BK16 and mono-, di- and tri-methylations of H3K4. To investigate whether there exists any difference in characteristics of nucleosome dynamics between regulatory regions and genomic regions, we separated the data above into two datasets, corresponding to chromosome III and promoter regions. For each dataset, we filtered out data of linker regions to keep only nucleosomal data. Each nucleosome was assigned either as *Well-positioned *if it stretched from 6 to 8 fragments or as *Delocalized *if it stretched more than 9 fragments. Nucleosomes which had no histone modification values or delocalized nucleosomes whose lengths were longer than 350 base pairs were also treated as noise and removed. After these preprocessing steps, the dataset of chromosome III contained 997 well-positioned nucleosomes and 154 delocalized nucleosomes, the dataset of promoter regions contained 995 well-positioned nucleosomes and 69 delocalized nucleosomes. These two datasets were used for further analysis.

### Method overview

**Figure 1:**
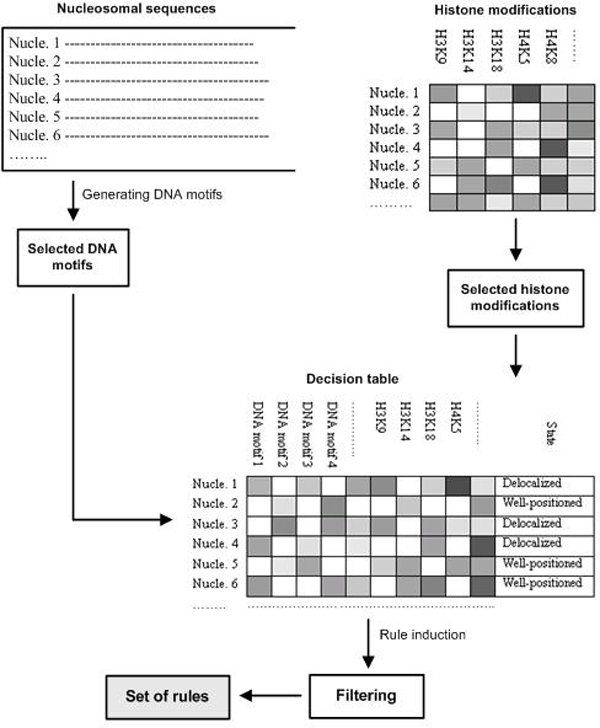
**Method overview**.

In this work we aim at characterizing how DNA sequences and histone modifications affect nucleosome dynamics. To this end, we propose a novel method that takes significant DNA motifs and histone modifications along with nucleosome states as the input for the rule induction system to infer patterns which may represent the dependence of nucleosome stability on these two factors. Figure 1 depicts the overview of our method. At first, DNA motifs, which might be signigicantly related to nucleosome stability, were extracted from nucleosomal sequences by applying two different approaches. The first one was to find potentially conserved motifs related to nucleosome states using WordSpy, the software that has been shown to outperform other competing motif finding methods on benchmark datasets. The second one was to find motifs which could serve as discriminative information for two states of nucleosomes using feature selection function of Gist software package [34]. Motifs were ranked based on their important levels identified by Fisher criterion. Significant histone modifications were also extracted by applying the same feature selection procedure as the second approach above. We then constructed a decision table from these significant DNA motifs and histone modifications (see Figure 1) and used it as the input for CN2-SD rule induction system (Section *Rule learning*) to produce a set of rules. Some filtering procedures were applied to remove uninteresting rules and keep rules which may meaningfully characterize nucleosome dynamics.

### Feature selection with Fisher criterion

Feature selection is a process of selecting a subset of relevant features available from the data that most contribute to distinguishing instances from different classes. In our method, significant sequence and histone modification features related to two states of nucleosomes, *Well-positioned *and *Delocalized*, were identified and ranked by their Fisher scores (or F-score in short). This is one of statistical criteria that is simple, effective and independent of the choice of classification method. Because our method only concentrated on identifying features with highly discriminative strength instead of building any concrete classifiers so we chose F-score as the selection criterion. The discriminative strength of each feature is defined as following:

Given a dataset *X *with two classes, denote instances in class 1 as *X*^1^, and those in class 2 as *X*^2^. Assume  is the average of the *jth *feature in *X*^*k*^, the F-score of the *jth *feature is:

Where

The numerator indicates the discrimination between two classes, and the denominator indicates the scatter within each class. The larger the F-score is, the more likely this feature is more discriminative.

### Rule learning

We consider this problem as a subgroup discovery problem and use a rule-based learning method for inducing rules. The problem of subgroup discovery can be defined as follows: given a population of individuals and a property of them, we are interested in finding population subgroups that are interesting with respect to the property of interest [26]. The induced rules usually have the form *Cond *→ *Class*, where *Class *is a value of the property of interest, and *Cond *is a conjunction of attribute-value pairs selected from the features describing the training instances. In our work, *Class *has two values, *Delocalized *and *Well-positioned*. Attributes are significant histone modifications and DNA motifs as described above (Section *Method overview*).

Among several available rule induction systems, CN2 is a rule induction system implementing the separate-and-conquer strategy [35]. It learns a rule set by iteratively adding rules one at a time. Examples covered by a rule are removed from the search space before learning the next rule to add to the rule set. This is repeated until all examples are covered by at least one rule in the rule set or some stopping criteria is satisfied. Finally, CN2 can induce a set of independent rules, where each rule describes a specific subgroup of instances. However, CN2 only induces the first few rules discovered are usually interesting. Subsequently induced rules are obtained from biased example subsets, i.e., subsets including only positive examples that are not covered by previously induced rules. In 2004, Lavrac and her colleagues developed an improvement of CN2 for subgroup discovery, so-called CN2-SD [26]. The CN2-SD generalizes the covering algorithm by introducing example weights. Initially, all examples have a weight of 1.0. However, the weights of examples covered by a rule will not be set to 0 (they are not removed as in CN2), but instead will be reduced by a certain factor. The resulting number of rules is typically higher than with CN2, since most examples will be covered by more than one rule. CN2-SD is, therefore, better in learning local patterns, since the influence of previously covered patterns is reduced, but not completely ignored. In order to evaluate the rules with higher generality, CN2-SD also uses a weighted relative accuracy heuristic as presented in Equation 3. The weighted covering strategy tends to find rules that explain overlapped subgroups of instances in the search space, so the weighted relative accuracy heuristic produces highly general rules that express the knowledge contained in one specific subgroup. For these reasons, we utilize the CN2-SD in the rest of this paper for finding rules.

### Rule filtering

Though the CN2-SD rule induction system uses a weighted covering strategy to restrict the redundancy of learned rules and guarantee the scanning of the whole search space, uninteresting rules are still produced [26,36]. Let us assume that our rule *r *has a form: *IF *[*Cond*] *THEN *[*ClassDistribution*]. Where *Cond *= [*motif*_1 _= *motifV al*_1 _∧ ... ∧ *motif*_*m *_= *motifV al*_*m*_*∧ histoneMod*_1 _= *hisV al*_1 _∧ ... ∧ *histoneMod*_*n *_= *hisV al*_*n*_] with *motif*_*i *_is a DNA motif, *motifV al*_*i *_is *enriched *or *low, histoneMod*_*j *_is one kind of histone modification and *hisV al*_*j *_is *hyper *or *neutral *or *hypo; ClassDistribution = [p, q] *with *p *and *q *are the number of *Well-positioned *and *Delocalized *nucleosomes covered by *r*, respectively. We used several heuristics to filter out unexpected rules: rules that cover less than 2 positive examples or *p*/(*p *+ *q*) < 0.8 if positive class is *Delocalized *and rules that cover less than 10 positive examples or *q*/(*p *+ *q*) < 0.8 if positive class is *Well-positioned *(Positive class is the class characterized by the rule).

## Competing interests

The authors declare that they have no competing interests.

## Authors' contributions

NTL and TBH defined the research problem. NTL and DHT designed the experiment. NTL, TBH and DHT drafted the manuscript. All authors contributed to and approved the final version of the manuscript.

## Note

Other papers from the meeting have been published as part of *BMC Bioinformatics* Volume 10 Supplement 15, 2009: Eighth International Conference on Bioinformatics (InCoB2009): Bioinformatics, available online at http://www.biomedcentral.com/1471-2105/10?issue=S15.

## Supplementary Material

Additional file 1 **The complete list of significant motifs on chromosome III given by WordSpy**. This file contains statistically significant motifs, with lengths from 2 bp to 6 bp, given by WordSpy while run on chromosome III. Click here for file

Additional file 2 **The complete list of significant motifs on promoter regions given by WordSpy**. This file contains statistically significant motifs, with lengths from 2 bp to 6 bp, given by WordSpy while run on promoter regions. Click here for file

Additional file 3 **The complete list of significantly discriminative motifs ranked by F-scores**. This file contains motifs of chromosome III and promoter regions ranked by their discriminative powers based on F-score. Click here for file

Additional file 4 **Rules characterizing nucleosome dynamics on chromosome III**. This file contains 60 rules characterizing nucleosome dynamics on chromosome III. Click here for file

Additional file 5 **Rules characterizing nucleosome dynamics on promoter regions**. This file contains 38 rules characterizing nucleosome dynamics on promoter regions. Click here for file
